# Mental Disorder—The Need for an Accurate Definition

**DOI:** 10.3389/fpsyt.2018.00064

**Published:** 2018-03-12

**Authors:** Diogo Telles-Correia, Sérgio Saraiva, Jorge Gonçalves

**Affiliations:** ^1^Clinica Universitaria de Psiquiatria e Psicologia, Faculty of Medicine, Universidade de Lisboa, Lisbon, Portugal; ^2^Faculty of Social and Human Sciences, IFILNOVA, Universidade Nova de Lisboa, Lisbon, Portugal

**Keywords:** mental disorders, definition and concepts, antipsychiatry, DSM classification, classification, homosexuality

## Abstract

There are several reasons why a definition for mental disorder is essential. Among these are not only reasons linked to psychiatry itself as a science (nosology, research) but also to ethical, legal, and financial issues. The first formal definition of mental disorder resulted from a deep conceptual analysis led by Robert Spitzer. It emerged to address several challenges that psychiatry faced at the time, namely to serve as the starting point for an atheoretical and evidence-based classification of mental disorders, to justify the removal of homosexuality from classifications, and to counter the arguments of antipsychiatry. This definition has been updated, with some conceptual changes that make it depart from the main assumptions of Spitzer’s original definition. In this article, we intend to review the factors that substantiated the emergence of the first formal definition of mental disorder that based all its later versions.

## Introduction

The existence of a formal definition of mental disorder remains essential for several reasons that include the following: 1) to know which diagnosis should or not be included in the classifications (1–3); 2) to separate areas of responsibility of the medical system from other societal systems; 3) to avoid dangerous medicalization of social problems; 4) to distinguish between pathological and normal; 5) to identify the conditions that, as a result of their negative consequences, implicitly have a call to action to the psychiatrists; 6) to identify the cases that justify societal recognition of the appropriateness of the sick role; 7) to understand which situations may prevent legal imputability; 8) to avoid false positives and other related problems such as overmedicalization, unnecessary labeling, wasted resources; and 9) to define psychiatry’s position as a special medical discipline (1–7).

The first formal definition of mental disorder was presented in DSM-III stemming from a deep conceptual review carried out by APA’s Committee on Nomenclature, headed by Spitzer. This definition was designed to address various needs psychiatry had at the time, notably to serve as a starting point for an atheoretical and evidence-based classification of mental disorders, to justify the removal of homosexuality from classifications, and to counter the arguments of antipsychiatry (according to which psychiatry was more oriented to social and ethical values rather than medical ones and could constitute a form of social control) (6, 7).

In recent times, this definition has been updated, with conceptual changes insufficiently discussed within psychiatry, and a consequent gradual detachment from Spitzer’s original definition. It is essential to reflect on the context in which this first formal definition of mental disorder emerges, and all the considerations that resulted therein, before making any updates to this definition.

This article intends to briefly review the factors that laid the foundations for the establishment of the first formal definition of mental disorder and which formed the basis for all its later versions.

### The Need for a Definition of Mental Disorder As a Starting Point for an Atheoretical and Evidence-Based Classification

Prior to the DSM-III, diagnostic classification was “based upon the best clinical judgment and experience of a committee and its consultants” [Ref. (5), p. 57]. The 1970s marked the beginning of a movement aimed at improving the quality of classification in psychiatry with various initiatives, including the development of the “Diagnostic Criteria for Use in Psychiatric Research”—DCPR (5) and the “Research Diagnostic Criteria” (RDC) (8). These initiatives represent the basis for the development of DSM-III nosological classification and criteria (6). The goal was to create systems with better validity for classifying mental disorders that did not depend, as in the past, on theoretical perspectives (psychodynamic, biological, etc). Spitzer believed that the starting point for any psychiatric classification should begin with the most atheoretical and value independent definition of mental disorder. Therefore, in the DSM-III “each of the mental disorders is conceptualized as a clinically significant behavioral or psychological syndrome or pattern that occurs in an individual and that is typically associated with either a painful symptom (distress) or impairment in one or more important areas of functioning (disability). In addition, there is an inference that there is a behavioral, psychological, or biological dysfunction (…)” [Ref. (9), p. 6].

Consistent with a descriptive and atheoretical structure, the DSM-III enhances the importance of the harm criteria (distress and disability) comparatively with the criteria of psychiatric/psychological dysfunction (generally linked to a specific theoretical point of view) (9).

### The Issue of Homosexuality and the Concept of Dysfunction

One of the contributing factors for the need to define mental disorder was an attempt not to include situations more related to cultural, moral, and religious values than to medical ones (which define what is harmful to the patient and should be treated) and which long undermined psychiatric classifications. Several such situations have been defined as mental disorders throughout history, such as, “drapetomania” (applied to American slaves who wanted to escape) and “sluggish schizophrenia” (applied to political dissidents in the Soviet Union). The case of homosexuality motivated a deep reflection within the APA on which situations to include as mental disorder or not.

According to Spitzer, the category of homosexuality did not make sense because one could not “insist on a label of sickness for individuals who insist that they are well (i.e., have ‘no subjective distress’) and who demonstrate no generalized impairment in social effectiveness” [Ref. (10), p. 1216].

In another document he refers “in reviewing the characteristics of the various mental disorders included in DSM-II, Spitzer concluded that, except for homosexuality, and perhaps other ‘sexual deviations,’ they all regularly caused subjective distress or were associated with generalized impairment in social effectiveness or functioning. It was proposed that the consequences of a condition, and not its etiology determined whether the condition should be considered a disorder” [Ref. (11), p. 16]. These arguments led Spitzer to privilege the criteria of harm (distress and disability) in detriment of criteria such as dysfunction, which was the main criterion for the definition of medical disease at the time.

The most defended model of disease in the 20th century was Boorse’s. According to this model, a disease corresponds to a dysfunction—alteration of natural functions resulting in reduced life expectancy and/or reproductive expectations (supposedly a value-free concept), emphasizing the importance of a dysfunction (biological or psychological) for the existence of a disease (12, 13, 14). Later, Wakefield agrees with Boorse’s dysfunction concept, arguing that these mechanisms may be physical or mental (12): “mental processes play important species-typical roles in human survival and reproduction, there is no reason to doubt that mental processes were naturally selected and have natural functions as Darwin himself often emphasized” [Ref. (12), p. 375]. But for Wakefield, the presence of dysfunction is insufficient to determine the presence of medical disturbance, the criteria of harm (distress–disability) must also be present, disagreeing with Boorse at this point (for whom biological or mental dysfunction suffices) (7). Both Boorse and Wakefield considered that the concept of dysfunction is independent of values and essential for the definition of any medical or psychiatric disorder.

Numerous criticisms have since arisen to the possibility of classifying certain psychological characteristics such as dysfunctions (7). Wakefield himself assumes this when stating that in some cases, such as homosexuality, it is difficult to assume the criterion of dysfunction (such as psychological alteration that interferes with the reproductive function), “in the contemporary context of overpopulation and widespread birth control among heterosexuals, the highest generally accepted normative goal of sexual-love relationships in our society is not reproduction *per se* but mutual interpersonal and sexual satisfaction” [Ref. (15), p. 676].

Thus, the most appropriate criteria for the definition of mental disorder and depending upon more universal values (than those associated with the definition of psychological dysfunction) would be those of distress and disability (7). As Gert and Culver stated “every society regards death, pain, disability and loss of pleasure as harms” [Ref. (16), p. 421].

Perhaps that is why Spitzer masterfully highlighted the presence of distress and disability criteria as priority in the definition of mental disorder in order to avoid and exclude dubious situations such as homosexuality, and other entities historically considered as mental disorders, alleging a psychological dysfunction assessed according to values (social, moral, and cultural) often masked as science. So Spitzer says about his definition of mental disorder that “these criteria avoid such terms as ‘dysfunction’… which themselves beg definition” [Ref. (1), p17]. Though he ends up using it (perhaps to not completely dissociate himself from the definition of medical disease valid at the time) referring “in addition, there is an inference that there is a behavioral, psychological, or biological dysfunction” [Ref. (9), p. 6], the universal criteria of distress and disability undoubtedly take supremacy in this definition.

### Antipsychiatry

Although antipsychiatry in the general sense of the term is as old as psychiatry (17), emerging in the 19th century, the movement known as “antipsychiatry” rose in the 1960s and 1970s. Associated with this movement were the names of Foucault, Szasz, Basaglia, Cooper, Laing, and others. Although there are several “antipsychiatries,” the common denominator is the fight against the psychiatric institution synthesized in the figure of the doctor and his power (18, 19). Criticism of repressive psychiatric power is the essential point of the movement, being the criticism of the concept of mental disorder and pharmacological therapies derivatives of this fundamental position.

In the book *Madness and Civilization*, Foucault presents the historical genesis of the concept of mental disorder, as we understand it today, which he does not separate from the psychiatric institution. That is to say, no knowledge about mental disorder is separated from its place of formation, the asylum. Asylum, in turn, follows a series of historical experiences that are predominantly of social control. Thus, finally, claiming that the science of psychiatry is essentially a knowledge seeking to justify the moral power of the physician. According to this view, psychiatrists forget this origin of their power and attribute it to objective knowledge obtained scientifically (20).

Szasz also considered the concept of mental disorder to be a myth (21). There is psychological suffering and life problems, but it will be a categorical mistake to consider them as “diseases” (22). Body and mind would belong to two orders of *being* qualitatively different. The body of medicine is explained by a mechanistic causality, and physiological diseases are lesions of organs, perfectly identified. The mind could not be explained by mechanical causes because it is intentionality and rationality. Thus, according to Szasz, one should not speak of mental “disorders” but of deviations from socially accepted norms and values. It would not be a question of the violation of the natural order, but of the social order (21).

Considering all these arguments, psychiatry had no alternative but to strengthen its status with a valid definition of mental disorder that would deviate from social, moral, and religious values. It should therefore be a priority to delimit the activity of psychiatry as a medical specialty, according to the main objective of medicine: the relief of the patient’s symptoms linked to distress and disability. Thus, distress and disability became the main criteria of this new definition of mental disorder. Spitzer himself preferred the name of disorder rather than disease highlighting that “there is no assumption that the organismic dysfunction or its negative consequences are of a physical nature,” because disease “often denotes a progressive physical disorder with known pathophysiology” [Ref. (1), p. 17], accepting a general basic difference between mental disorders and medical disease.

Conversely, giving a primordial role to the criteria of harm could also divert the focus of mental disorder, from the doctor (his values and power) to the patient and his needs arising from the suffering he bears.

## Discussion

The first formal definition of mental disorder appears in DSM-III as a result of a deep conceptual review. This definition emerged to meet various needs of psychiatry at that time, in particular to serve as a starting point for an evidence-based and atheoretical classification of mental disorder and to justify the removal of homosexuality from classifications and counter the arguments of antipsychiatry. A definition was elaborated in which the main condition for a mental disorder to be present was the presence of the criteria of distress and disability (less permeable to theoretical differences and to moral, cultural, and religious values than the concept of psychiatric or psychological dysfunction).

The criteria of harm (distress and disability) remained as paramount in the definition of mental disorder in DSM-IV and the importance of these criteria also led them to make part of the specific diagnostic criteria for most disorders listed in DSM-IV.

Nevertheless, in DSM-5 a major and barely discussed change occurred, the concept of dysfunction takes precedence, appearing at the beginning of the definition, possibly being considered its main criterion:

A mental disorder is a syndrome characterized by clinically significant disturbance in an individual’s cognition, emotion regulation, or behavior that reflects a dysfunction in the psychological, biological, or developmental processes underlying mental functioning. Mental disorders are usually associated with significant distress or disability in social, occupational, or other important activities… [Ref. (23), p. 20].

Harm criteria are no longer a basic requirement, but a frequent occurrence that might or not be present.

This could lead to the inclusion (in psychiatric diagnostic manuals) of situations that are not associated with distress and disability as happened in the past, potentially re-exposing psychiatry to the danger that entities considered psychological or biological dysfunctions, according to certain theoretical currents (easily permeable to moral and social values), may be considered mental disorders.

The problem of considering a mental disorder to be mainly a dysfunction (as in DSM-5) may arise in both perspectives: as a biological dysfunction or as a psychological dysfunction.

Several problems arise regarding the possibility of considering mental disorders as synonymous of biological dysfunction. Psychiatric disorders are not natural kinds directly visualized and discriminated by neuroimaging tests. Psychiatric disorders are “social constructs” that do not exist independently of human effort (6, 24, 25). The evaluation of what is pathological or not in psychiatry is related to 1) comprehensibility (whether or not the mental state/behavior is comprehensible given the sociocultural context of the patient), 2) adaptability (adaptive or non-adaptive in the context of the patient), and 3) connection to distress and disability (whether or not they cause distress or disability) (26). The latter being the most universal criteria (7, 24). The abovementioned criteria define the presence or absence of mental symptoms or disorder that is primary or secondary to a physical dysfunction. As an example, in the case of a brain tumor that subsequently induces depressive symptoms, clinical depression is assessed through clinical criteria (comprehensibility, adaptability, and harm inducing). Mental manifestations, regardless of the physical or neurological core, only represent a mental disorder if they are regarded as inadequate, non-adaptative, or causing harm (considering the sociocultural background and circumstances of the patient). That cannot mean that we cannot try to find the physical or neurological correlates of these mental manifestations. However, in psychiatry, separation of disordered from non- disordered is not dependent upon neurological biomarkers. This means that clinical concepts are precursors to biological concepts. Thus, mental disorder cannot only be defined by a physical or biological dysfunction (27).

Conversely, it is also controversial to define mental disorder through a psychological dysfunction. As mentioned by Fullford, dysfunction as the concept of failure of a mechanism to determine a natural function is a concept inextricably linked to values. For biological issues and medical values (what is considered useful or not to the organism by specific societies) change over time (28, 29). Additionally, there is much less consensus about the concept of psychological dysfunction than that of biological dysfunction, due to an insufficient knowledge about the psychological processes. Thus, recognizing the dysfunctional aspects of psychological mechanisms is harder (30). Notwithstanding, mental functions are directly bound to a social role that physical functions are not, meaning that the former are much more linked to social and cultural values (30).

Moreover, the definition of dysfunction of psychological mechanisms, as a failure of internal mechanisms to perform their functions as designed by nature, traces an artificial boundary between what is natural (innate), as opposed to social (cultivated) (22). Additionally, “human behavior is also socially designed and the relative contributions of biology and social rules are complex and interwoven, not easy to tease apart” [Ref. (25), p. 124].

Kirmayer adds that: “there is little consensus on what our psychological systems are for and many evolutionary psychologists argue that we have evolved to be able to adapt to situations rather than to have fixed or specific functions. Any change in culture will change the fitness of specific psychological, traits, give new meaning and purpose to biological functions, and change their boundaries and interdependence. Beyond relatively simple physiological functions it is impossible to identify what psychological systems or functions are for in any universal sense” [Ref. (31), pp. 18–19].

Furthermore, psychiatric disorders could be caused by distinct situations, instead of disruption function (e.g., defensive/coping strategies, design/environment mismatches, maladaptive-looking phenotypes that may be adaptive; highly evolved learning capacities leading to maladaptive behavior) (25).

## Conclusion

The difficulties inherent to the use of the concept of dysfunction to define a psychiatric disorder are not only a problem of validity in psychiatry but may also make psychiatry permeable (again) to the typical criticisms of antipsychiatry which claimed that psychiatric disorders were more linked to values associated with culture-specific social and political ideologies rather than medical values (which identify harmful situations to the patient and in need of treatment). Spitzer attempted to circumvent these issues by making distress and disability (revealed at the patients’ level) the main defining criteria of mental disorders. These are concepts that are closer to the patient than to the psychiatrist and that are loaded with more universal values (it is consensual that distress and disability are negative for the patient and deserve to be relieved). On the other hand, as mentioned, the determination of biological or even psychological dysfunction in most psychiatric disorders is difficult, controversial, and usually the result of influenceable theoretical currents.

In essence, the first definition of mental disorder resulted from a rigorous conceptual analysis. We should continue to promote critical reviews and conceptual analysis on this topic which can debate the problems that it entails and the dangers that an apparently harmless change to the definition of mental disorders (such as that which was taken in DSM 5) can bring toward psychiatry.

## Author Contributions

DT-C conceived and designed research. DT-C and SS wrote the first draft of the manuscript. JG did critical revision for important intellectual content. The manuscript has been read and approved by all the authors. There are no other persons who satisfied the criteria for authorship but are not listed. We further confirm that the order of authors listed in the manuscript has been approved by all of us.

## Conflict of Interest Statement

The authors declare that the research was conducted in the absence of any commercial or financial relationships that could be construed as a potential conflict of interest.
